# Comparison of Handmade Endoloop Versus Polymeric Endoclip for Stump Closure in Laparoscopic Appendectomy

**DOI:** 10.7759/cureus.16302

**Published:** 2021-07-10

**Authors:** Ahmet Erdoğan, Ahmet Türkan

**Affiliations:** 1 General Surgery, Kahramanmaraş Elbistan State Hospital, Kahramanmaraş, TUR

**Keywords:** complication, appendix stump, handmade endoloop, polymer clips, laparoscopic appendectomy

## Abstract

Background

Stump closure is an important stage of laparoscopic appendectomy. This study aimed to establish whether the handmade endoloop or polymeric endoclip method was more effective for stump closure in laparoscopic appendectomy.

Methods

The study included 76 patients who underwent laparoscopic appendectomy between October 2017 and January 2019. Patients’ demographic characteristics, duration of surgery, length of hospital stay, and any complications were retrospectively recorded from files. Patients were divided into two groups according to stump closure method as polymeric endoclip and handmade endoloop.

Results

Among the patients, 59.2% (n = 45) were male and 40.8% (n = 31) were female. For stump closure, the polymeric endoclip method was used in 37 patients (48.7%) and the handmade endoloop method in 39 patients (51.3%). The two groups were not significantly different in terms of age and appendix diameter ( p=0.408, p=0.218). A total of four patients (5.3%) developed wound infection, including three from the handmade endoloop group and one from the polymeric endoclip group. One patient (1.3%) in the polymeric endoclip group developed ileus. The two groups were also not significantly different in terms of complications (p = 1.000).

Conclusion

We conclude that both stump closure methods are safe, and the more easily accessible handmade endoloop method can be performed reliably in all hospitals, including secondary healthcare facilities such as small hospitals.

## Introduction

Acute appendicitis is one of the most common causes of emergency surgical intervention. Diagnosis is usually established based on patient history and physical examination and confirmed by laboratory and imaging methods [1-2]. Ultrasonography (US) and computed tomography (CT) of the abdomen are imaging methods commonly used for diagnosis [3-4]. Laparoscopic appendectomy (LA) is an alternative to open appendectomy with increasing application in Europe and the United States. The European Endoscopic Surgery Association recommends LA [5].

Laparoscopic appendectomy has several advantages over open appendectomy, including reduced length of hospital stay, pain, wound infection, and better cosmetic outcomes [6]. The risk of appendiceal stump leak and subsequent abdominal sepsis are feared complications of LA [7]. Various stump closure techniques have been described in LA; the most common include endoloop, suture, stapler, and endoclip methods. As of yet, there is no consensus on the optimal treatment method [5].

This study aimed to compare the effectiveness of the handmade endoloop and polymeric endoclip methods that we commonly employ in our clinic for stump closure in LA.

## Materials and methods

The study included patients who underwent LA in the general surgery clinic of a secondary public hospital in Turkey between October 2017 and January 2019. Patients’ demographic characteristics, duration of surgery, length of hospital stay, and any complications were retrospectively recorded from files. Patients were divided into two groups according to stump closure method as polymeric endoclip and handmade endoloop. For the handmade endoloop, a 2/0 Vicryl (polyglactin) suture was used. All operations were performed by the same surgical team. All patients were preoperatively administered 1 g of i.v. cefazolin for prophylaxis. In all operations, the mesoappendix was dissected using LigaSure (Covidien, Dublin, Ireland).

Patients aged <18 years and those with perforated appendicitis were excluded from the study. The study was granted ethical approval by the Malatya Inonu University Clinical Research Ethics Committee (date April 24, 2019, decision number 2019/89).

Statistical analysis

Data were analyzed using IBM SPSS Statistics for Windows, Version 21.0 (Released 2012; IBM Corp., Armonk, NY). Conformity of the data to normal distribution was evaluated by the Shapiro-Wilk test. The data were not normally distributed; therefore, numerical data were expressed using medians and interquartile range (IQR) and categorical data were expressed using numbers and percentages. The two groups were compared using the Mann-Whitney test for numerical data and the chi-square test for categorical data. Values of p<0.05 were considered statistically significant.

## Results

The study included 76 patients (45 men and 31 women). The median age was 30.5 years (IQR:20). The diagnosis was confirmed by US examination in 15 patients (19.7%) by CT examination in 38 patients (50%) and by US and CT in 23 patients (30.3%). A polymeric endoclip was used for stump closure in 37 patients (48.7%) and a handmade endoloop in 39 patients (51.3%). No complications occurred in 71 (93.4%) patients. A total of four patients (5.3%) developed wound infection, including three from the handmade endoloop group and one from the polymeric endoclip group. One patient (1.3%) in the polymeric endoclip group developed ileus. None of the subjects developed an appendiceal stump leak.

The two groups were not different in terms of gender (p = 0.647) (Table 1).

**Table 1:** Comparison of treatment methods by gender

			Gender		p
			Male	Female	
Closure method	Polymeric endoclip	n	23	14	0.647
		%	62.2	37.8	
	Handmade endoloop	n	22	17	
		%	56.4	43.6	

The two groups were not different in terms of imaging methods (p = 0.163) (Table 2).

**Table 2:** Comparison of treatment methods by imaging method

			Imaging			p
			US	CT	US + CT	
Closure method	Polymeric endoclip	n	6	16	15	0.163
		%	16.2	43.2	40.5	
	Handmade endoloop	n	9	22	8	
		%	23.1	56.4	20.5	

The two groups were not significantly different in terms of age and appendix diameter measured radiologically (p=0.408, p=0.218) (Table 3).

**Table 3:** Distribution of age and appendix diameter according to treatment methods

	Polymeric endoclip (n = 37)	Handmade endoloop (n = 39)	p
Age (years)	33 (range: 18-74)	27 (range: 18-65)	0.408
Appendix diameter (mm)	9 (range: 7-13)	10 (range: 7-14)	0.218

Complication rates were similar between groups. While in the polymeric endoclip method, complications developed in two patients (5.4%), in the handmade endoloop method, complications developed in three patients (7.7%) (Table 4).

**Table 4:** Comparison of treatment methods according to complications

			Complication	
			No	Yes
Closure method	Polymeric endoclip	n	35	2
		%	94.6	5.4
	Handmade endoloop	n	36	3
		%	92.3	7.7

The two groups were not different in terms of duration of operation or length of hospital stay (p=0.307, p=0.597). The median duration of operation was 40 minutes (range: 17-85) in the polymeric endoclip group and 40 minutes (range: 27-63) in the handmade endoloop group. The median length of hospital stay was one day (range: 1-7) in the polymeric endoclip group and one day (range: 1-2) in the handmade endoloop group (Table 5).

**Table 5:** Distribution of duration of operation and length of hospital stay according to treatment methods

	Polymeric endoclip (n = 37)	Handmade endoloop (n = 39)	p
Length of hospital stay (days)	1 (range: 1-7)	1 (range: 1-2)	0.597
Duration of operation (minutes)	40 (range: 17-85)	40 (range: 27-63)	0.307

## Discussion

In LA, various stump closure techniques have been described, including intra- and extracorporeal knots, staplers, polymeric endoclips, and endoloops. Numerous studies compared these techniques to establish an ideal method [8]. Endo staplers are preferable for stump closure when the stump is wide and necrotic; however, this method is not always feasible due to the high cost and requirement of a wide trocar [9].

Studies show that LA is more costly than open appendectomy. Therefore, researchers have investigated alternatives to reduce the costs and reported that this was possible with alternative stump closure methods such as the handmade endoloop technique [10]. In their study of 98 patients, Yıldız et al. reported that stump closure with a handmade endoloop was cost-effective and safe [11].

Antoniou et al. reported that stump closure by suture was superior to other methods in terms of wound infection [5]. In our study, three patients (7.7%) from the handmade endoloop group and one patient (2.7%) from the polymeric endoclip group developed wound infection. We did not find the two methods to be statistically different in terms of wound infection.

Nadeem et al. compared extracorporeal suture and metallic endoclip in terms of postoperative ileus and found no statistically significant difference between the two groups. One patient in the metallic endoclip group and two patients from the extracorporeal suture group developed ileus [12]. In our study, one patient from the polymeric endoclip group developed ileus.

Şimşek et al. compared a polymeric endoclip and an endoloop in stump closure in LA and reported that the polymeric clip facilitated the surgical technique and reduced operation time [8].

Partecke et al. stated that the Hem-o-lok clip is safe and practical, but if the appendiceal stump is inflamed and wide, closure safety may be problematic [13].

Bali et al. compared intracorporeal ligation with an endoloop and stated that the operation time was reduced with the endoloop [14]. Ateş et al. compared the use of an intracorporeal knot with a titanium endoclip in the closure of the appendiceal stump. The mean operation time was 41.27 minutes in the endoclip group and 62.81 minutes in the knot group [15]. Lucchi et al. compared appendiceal stump closure with a Hem-o-lok clip and an endoloop. Mean operation time was 40.5 minutes for the endoloop and 36.4 minutes for the Hem-o-lok clip. The length of hospital stay was similar for the two groups, and there was no statistically significant difference in terms of complications [16].

In our study, the polymeric endoclip and handmade endoloop groups were not different in terms of duration of operation (p = 0.307), hospital stay (p = 0.597), or postoperative complications (p = 1.000).

The limitations of our study are its retrospective nature and the relatively small number of patients due to being conducted in a small medical center.

## Conclusions

We conclude that both stump closure methods were safe. Moreover, the more easily accessible and cost-effective handmade endoloop method can be performed reliably in all hospitals, including secondary healthcare facilities such as small hospitals.

## References

[1] Çetinkaya E, Erdoğan A, Akgül Ö, Çelik C, Tez M (2015). High serum cancer antigen 125 level indicates perforation in acute appendicitis. Am J Emerg Med.

[2] Hakkoymaz H, Nazik S, Seyithanoğlu M, Güler Ö, Şahin AR, Cengiz E, Yazar FM (2019). The value of ischemia-modified albumin and oxidative stress markers in the diagnosis of acute appendicitis in adults. Am J Emerg Med.

[3] Rud B, Vejborg TS, Rappeport ED, Reitsma JB, Wille-Jørgensen P (2019). Computed tomography for diagnosis of acute appendicitis in adults. Cochrane Database Syst Rev.

[4] Ashkenazi I, Zeina AR, Olsha O (2020). Early ultrasound in acute appendicitis avoids CT in most patients but delays surgery and increases complicated appendicitis if nondiagnostic - a retrospective study. Am J Surg.

[5] Antoniou SA, Mavridis D, Hajibandeh S (2017). Optimal stump management in laparoscopic appendectomy: a network meta-analysis by the Minimally Invasive Surgery Synthesis of Interventions and Outcomes Network. Surgery.

[6] Yaghoubian A, Kaji AH, Lee SL (2012). Laparoscopic versus open appendectomy: outcomes analysis. Am Surg.

[7] Agalar C, Derici S, Çevlik AD (2019). Do the stump knotting technique and specimen retrieval method effect morbidity in laparoscopic appendectomy?. Ulus Travma Acil Cerrahi Derg.

[8] Şimşek O, Bilgin İA, Uludağ S, Dal F, Velidedeoğlu M, Sarıbeyoğlu K, Pekmezci S (2017). Comparison of endoloop and polymer locking clip in ligating appendiceal stump during laparoscopic appendectomy. Laparosc Endosc Surg Sci.

[9] Mayir B, Ensari CÖ, Bilecik T, Aslaner A, Oruç MT (2015). Methods for closure of appendix stump during laparoscopic appendectomy procedure. Ulus Cerrahi Derg.

[10] Mayir B, Bilecik T, Ensari CO, Oruc MT (2014). Laparoscopic appendectomy with hand-made loop. Wideochir Inne Tech Maloinwazyjne.

[11] Yildiz F, Terzi A, Coban S, Zeybek N, Uzunkoy A (2009). The handmade endoloop technique. A simple and cheap technique for laparoscopic appendectomy. Saudi Med J.

[12] Nadeem M, Khan SM, Ali S, Shafiqe M, Elahib MW, Abdullah F, Hussain I (2016). Comparison of extra-corporeal knot-tying suture and metallic endo-clips in laparoscopic appendiceal stump closure in uncomplicated acute appendicitis. Int J Surg Open.

[13] Partecke LI, Kessler W, von Bernstorff W, Diedrich S, Heidecke CD, Patrzyk M (2010). Laparoscopic appendectomy using a single polymeric clip to close the appendicular stump. Langenbecks Arch Surg.

[14] Bali İ, Karateke F, Özyazıcı S (2015). Comparison of intracorporeal knotting and endoloop for stump closure in laparoscopic appendectomy. Ulus Travma Acil Cerrahi Derg.

[15] Ates M, Dirican A, Ince V, Ara C, Isik B, Yilmaz S (2012). Comparison of intracorporeal knot-tying suture (polyglactin) and titanium endoclips in laparoscopic appendiceal stump closure. A prospective randomized study. Surg Laparosc Endosc Percutan Tech.

[16] Lucchi A, Berti P, Grassia M, Siani LM, Gabbianelli C, Garulli G (2017). Laparoscopic appendectomy: Hem-o-lok versus endoloop in stump closure. Updates Surg.

